# Delayed onset Aspergillus niger keratitis post-DALK associated with bandage contact lens: a case report highlighting the importance of vigilant postoperative care and management

**DOI:** 10.3205/oc000260

**Published:** 2025-11-21

**Authors:** Dharamveer Singh Choudhary, Nolan Rodrigues, Jeba Shaheen, Sidhya Choudhary, Ajay Dhakad, Bhuvanesh Sukhlal Kalal

**Affiliations:** 1 Department of Ophthalmology, Swai Man Singh Medical College and Hospitals, Jaipur, Rajasthan, India; 2 Department of Pharmacology and Nutritional Sciences, College of Medicine, University of Kentucky, Lexington, Kentucky, USA

**Keywords:** lamellar keratoplasty, Aspergillus niger, bandage contact lens, corneal ulcer, fungal infection

## Abstract

We present the case of an 18-year-old male who developed a black-colored growth on a bandage contact lens and cornea nine months after undergoing lamellar keratoplasty for advanced keratoconus in his left eye. The growth was identified as Aspergillus niger, which was successfully treated following its removal and microbiological examination.

## Case description

An 18-year-old male, with a known history of advanced keratoconus in the left eye, underwent deep anterior lamellar keratoplasty (DALK) nine months prior. The patient had been compliant with his postoperative follow-ups, which had been uneventful until this point. However, he presented to the clinic with an acute complaint of a blackish growth in his left eye. The symptoms included a sudden onset of redness, excessive tearing (epiphora), and mild ocular discomfort. There was no history of recent trauma, systemic illness, or any other significant health events. The patient recalled a bandage contact lens being applied two months earlier as part of his postoperative care, but he did not report any issues with its use until this visit.

**Figure 1:**
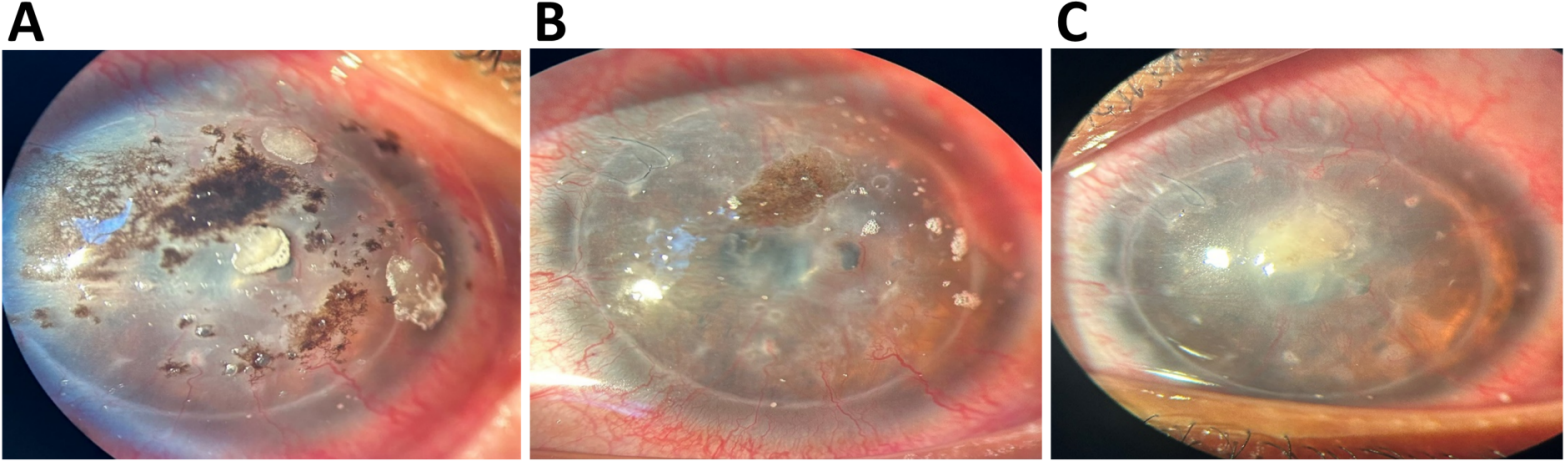
Clinical course and treatment response of Aspergillus niger keratitis following DALK. Blackish growth was observed under the bandage contact lens at presentation (A). Corneal ulcer with blackish growth covering the base following removal of the bandage contact lens (B). Clinical appearance one week after initiating treatment (C)

On slit-lamp examination, a dense, black-colored growth was observed under surface of the bandage contact lens (Figure 1 (Fig. 1)). There was also significant conjunctival congestion, indicating inflammation. Upon removal of the contact lens, a corneal ulcer with a black pigmented base was revealed. The ulcer was approximately 3 mm in diameter, located in the central cornea, and had well-defined margins. Additionally, superficial circumferential vascularization was noted around the cornea, which is often indicative of chronic irritation or an ongoing inflammatory process. The anterior chamber was quiet, with no signs of hypopyon or fibrin, and intraocular pressure was within normal limits.

Visual acuity in the affected eye had deteriorated to 6/60, a significant reduction from the patient’s baseline postoperative vision. Given the alarming appearance of the corneal lesion and the potential for severe visual loss, a thorough diagnostic workup was initiated immediately.

Corneal scrapings were obtained from the ulcer base and the surrounding corneal tissue for microbiological evaluation. These samples were subjected to Gram staining, potassium hydroxide (KOH) mount, and cultures on Sabouraud dextrose agar to identify potential bacterial or fungal pathogens.

While awaiting culture results, empirical treatment was initiated based on the clinical suspicion of fungal keratitis. The patient was started on intensive topical antifungal therapy, including natamycin 5% eye drops every two hours and voriconazole 1% eye drops every two hours. An additional antifungal agent, itraconazole 1% ointment, was prescribed for nighttime application to ensure continuous drug presence on the corneal surface. To manage inflammation and prevent synechiae, atropine 1% eye drops were administered three times daily. Systemic antifungal therapy with oral ketoconazole 200 mg twice daily was also started to enhance the treatment efficacy. Oral doxycycline 100 mg twice daily was prescribed to reduce the risk of corneal melting, and vitamin C 500 mg once daily was included to support corneal healing.

The Gram stain of the corneal scrapings did not reveal any bacterial organisms, but the KOH mount showed the presence of numerous branched fungal hyphae, confirming the diagnosis of fungal keratitis. Three days later, the culture results confirmed the growth of Aspergillus niger, a known pathogenic fungus associated with contact lens-related keratitis.

The patient was advised on the importance of adherence to the prescribed treatment regimen and scheduled for close follow-up visits. During these visits, gradual improvement in corneal clarity and reduction in the size of the ulcer was noted, with a corresponding improvement in visual acuity. The patient was also counseled extensively on the importance of maintaining strict eye hygiene, using protective eyewear, and avoiding environments that could expose him to fungal pathogens in the future.

## Discussion

The use of hydrophilic contact lenses following keratoplasty is widely recognized for its multifaceted benefits [1]. Primarily, these lenses offer postoperative pain relief by shielding the corneal surface from mechanical trauma caused by the eyelids and the external environment. Additionally, by providing a smooth refractive surface, they help to minimize postoperative corneal irregularities, which can significantly improve visual acuity in patients who have undergone lamellar keratoplasty.

However, despite these advantages, the use of bandage contact lenses can also predispose patients to microbial keratitis, a serious complication characterized by the infection of the corneal stroma [2]. While bacterial keratitis is more commonly reported, fungal infections, though less frequent, are particularly challenging to manage due to their sluggish nature and the difficulty in early diagnosis [3]. Fungal keratitis is often associated with extended wear of contact lenses, especially in warm and humid climates, where fungi such as Candida, Aspergillus, and Fusarium thrive [4].

In this case, the late onset of fungal keratitis nine months after keratoplasty highlights the insidious nature of fungal infections. Aspergillus niger, the pathogen identified in this patient, is a common environmental fungus that can contaminate contact lenses and solutions. Once established, fungal keratitis can lead to significant corneal destruction if not promptly diagnosed and treated [5]. The typical clinical signs include a corneal ulcer with feathery edges, satellite lesions, and, in some cases, a pigmented appearance, as seen in this patient.

Management of fungal keratitis involves prompt initiation of antifungal therapy. Topical natamycin remains the first-line treatment for filamentous fungal keratitis due to its broad-spectrum activity and ability to penetrate the corneal stroma [6]. In topical and systemic forms, Voriconazole has emerged as an effective alternative, especially for infections caused by Aspergillus species [7]. Oral antifungals such as ketoconazole or itraconazole may be added for severe cases or those not responding to topical therapy [8].

This case emphasizes the importance of patient education in the postoperative period, particularly regarding maintaining eye hygiene and the risks associated with prolonged contact lens wear. Patients should be advised to adhere strictly to follow-up schedules, as early detection of complications like fungal keratitis can prevent severe visual impairment. Furthermore, protective eyewear should be recommended for patients in environments conducive to fungal growth, such as dusty or humid conditions.

## Conclusion

This case underscores the importance of preventive measures in post-keratoplasty care, including regular contact lens changes, patient education on eye hygiene, the use of protective eyewear, timely medication application, and regular follow-ups to detect and treat complications early.

## Notes

### Patient consent

The authors certify they have obtained all appropriate patient consent. 

### Competing interests

The authors declare that they have no competing interests.
